# Causal relationship between multiple sclerosis and cortical structure: a Mendelian randomization study

**DOI:** 10.1186/s12967-024-04892-7

**Published:** 2024-01-20

**Authors:** Dongren Sun, Rui Wang, Qin Du, Ying Zhang, Hongxi Chen, Ziyan Shi, Xiaofei Wang, Hongyu Zhou

**Affiliations:** Department of Neurology, West China Hospital, Sichuan University, Guo Xuexiang #37, Chengdu, 610041 China

**Keywords:** Multiple sclerosis, Cortical structure, Mendelian randomization

## Abstract

**Background:**

Observational studies have suggested an association between multiple sclerosis (MS) and cortical structure, but the results have been inconsistent.

**Objective:**

We used two-sample Mendelian randomization (MR) to assess the causal relationship between MS and cortical structure.

**Methods:**

MS data as the exposure trait, including 14,498 cases and 24,091 controls, were obtained from the International Multiple Sclerosis Genetics Consortium. Genome-wide association study (GWAS) data for cortical surface area (SAw/nw) and thickness (THw/nw) in 51,665 individuals of European ancestry were obtained from the ENIGMA Consortium. The inverse-variance weighted (IVW) method was used as the primary analysis for MR. Sensitivity analyses were conducted to evaluate heterogeneity and pleiotropy. Enrichment analysis was performed on MR analyses filtered by sensitivity analysis.

**Results:**

After IVW and sensitivity analysis filtering, only six surviving MR results provided suggestive evidence supporting a causal relationship between MS and cortical structure, including lingual SAw (*p* = .0342, beta (se) = 5.7127 (2.6969)), parahippocampal SAw (*p* = .0224, beta (se) = 1.5577 (0.6822)), rostral middle frontal SAw (*p* = .0154, beta (se) = − 9.0301 (3.7281)), cuneus THw (*p* = .0418, beta (se) =  − 0.0020 (0.0010)), lateral orbitofrontal THw (*p* = .0281, beta (se) = 0.0025 (0.0010)), and lateral orbitofrontal THnw (*p* = .0417, beta (se) = 0.0029 (0.0014)). Enrichment analysis suggested that leukocyte cell-related pathways, JAK-STAT signaling pathway, NF-kappa B signaling pathway, cytokine-cytokine receptor interaction, and prolactin signaling pathway may be involved in the effect of MS on cortical morphology.

**Conclusion:**

Our results provide evidence supporting a causal relationship between MS and cortical structure. Enrichment analysis suggests that the pathways mediating brain morphology abnormalities in MS patients are mainly related to immune and inflammation-driven pathways.

**Supplementary Information:**

The online version contains supplementary material available at 10.1186/s12967-024-04892-7.

## Introduction

Multiple sclerosis (MS) is a multifactorial central nervous system autoimmune disease that leads to demyelination and axonal loss, with a male-to-female ratio of 1:3 [1]. MS affects over 2 million people, imposing a significant global health burden [2]. Some studies have reported that approximately 40–70% of MS patients experience cognitive dysfunction [3, 4], and up to 60% have neuropsychiatric symptoms [5, 6]. These neuropsychiatric abnormalities may be related to changes in brain connectivity. Accumulating evidence suggests that gray matter structures are widely affected in MS patients [7–9]. A 1-year follow-up study of 75 relapsing–remitting MS (RRMS) and 11 progressive MS (PMS) patients showed that RRMS patients had thinning of the frontal and temporal cortices, while PMS patients had a general decrease in cortical thickness (TH) [10]. Several other studies have confirmed that RRMS patients exhibit a decrease in average TH across the entire brain [11–13]. Nygaard et al. found that RRMS patients had similar cortical surface area (SA) to healthy controls [14], but another study provided evidence supporting a decrease in central anterior SA in MS patients [15].

However, these reports of the association between MS and brain structure are observational, and the conclusions are not consistent. Moreover, these studies are vulnerable to confounding factors and reverse causality, making causal inference difficult using traditional observational epidemiology. More importantly, the extent to which MS affects cortical structures and the underlying mechanisms remain unclear. Therefore, a tailored approach to exploring the causal relationship between MS and the cortical brain structure is crucial.

Mendelian randomization (MR) is a widely used approach in the study of neurological disorders, which employs single nucleotide polymorphisms (SNPs) as instrumental variables (IVs) to explore causal relationships between exposures and outcomes [16–19]. MR minimizes confounding and avoids reverse causation [17]. The fundamental principle in mitigating confounding factors within MR studies is rooted in the random allocation of genetic variations during meiosis, ensuring that one trait is typically unrelated to others. This method serves to avoid reverse causation, given that the genetic variations utilized to represent the impact of exposure remain unaltered by the occurrence and progression of outcomes [17]. There are three key assumptions in MR analysis. First, the genetic variation used as IVs should be associated with the risk factor of interest. Second, the genetic variation used should not be associated with potential confounders. Third, the selected genetic variation should only affect the outcome through the risk factor [20]. Here, we conducted a two-sample MR study using publicly available genome-wide association study (GWAS) data to determine the causal relationship between MS and brain structure.

## Methods

### Data sources and genetic instruments

#### Participants

We obtained MS GWAS data from the International Multiple Sclerosis Genetics Consortium (IMSGC). The dataset comprises 38,589 individuals of European ancestry, including 14,498 cases and 24,091 healthy controls. Disease diagnosis was conducted by neurologists familiar with MS, following widely recognized diagnostic criteria [21–23]. Disease severity was assessed using the Expanded Disability Status Score (EDSS) [24] and the Multiple Sclerosis Severity Score (MSSS) [25]. The overall age of onset for MS is 33.1 years. The study identified 48 new susceptibility loci for MS, highlighting the role of NF-kappa B in the disease's pathobiology. In addition, the overlap between MS and other autoimmune diseases was calculated using an immune chip analysis. Approximately 22% of the MS signals overlapped with at least one other autoimmune disease, primarily including inflammatory bowel disease (~ 9.1%), primary biliary cirrhosis (~ 9.1%), Crohn’s disease (~ 9.1%), ulcerative colitis (~ 7.3%), celiac disease (~ 4.5%), rheumatoid arthritis (~ 4.5%), and autoimmune thyroid disease (~ 2.7%) [26]. For further details on this study, please refer to the original publication [26].

#### Brain cortical structure

The summary-level data on the cortical structure is derived from the work of the Enhancing NeuroImaging Genetics through Meta-Analysis Consortium (ENIGMA)-Genetics working group. Grasby et al. utilized magnetic resonance imaging data from 51,665 individuals to investigate the cortical SA and TH [27]. The GWAS meta-analysis identified loci that affect regional SA near genes involved in the Wnt signaling pathway. Specifically, the study divided cortical structures into 34 functionally specialized regions based on the Desikan-Killiany atlas, and regional SA and TH were analyzed with and without whole-brain weighting (w indicates weighted regions, while nw indicates unweighted regions) [27]. Additional details can be found in the original research study [27]. Therefore, we utilized this GWAS data to analyze the causal effects of MS on both whole-brain and the 34 functional regions' cortical SA and TH, resulting in a total of 138 analyses.

#### Selection of instrumental variables

Based on the basic assumptions of MR, we first extracted MS-related IVs with a threshold of P < 5E−8. Second, we clumped the IVs based on the European 1000 Genomes Project (clumped R^2^ < 0.001, window size = 10 Mb). Third, proxy genetic variants were used when no corresponding SNPs were available (LD R^2^ threshold was set at 0.8). Fourth, we homogenized the SNPs and removed palindromic SNPs. To ensure the IVs had strong statistical power, we selected SNPs with an F-statistic greater than 10. We used the formula R^2^ * (N−k−1)/[ (1−R^2^) * k] to calculate the F-statistic, where N is the sample size of MS, k is the number of SNPs, and R^2^ is the proportion of MS variation explained by each SNP. R^2^ was calculated by the formula: 2* beta^2^* (1-eaf) * eaf, where eaf is the allele frequency of the effect, and beta is the estimate of the genetic effect of the variant on MS [28, 29]. Our MR study followed the STROBE-MR Statement guidelines [30].

### MR analysis and sensitivity analysis

The inverse-variance weighted (IVW) method with random effects is used as the main approach for MR analysis. The IVW approach combines the Wald ratio estimates of each SNP to obtain an overall estimate of the causal effect. Although IVW allows for heterogeneity [20, 31], it is susceptible to pleiotropic bias [32, 33]. MR-Egger, weighted median, simple mode, and weighted mode methods rely on assumptions different from those of IVW and are relatively robust to horizontal pleiotropy, which can complement IVW to make MR estimates more reliable [34]. Cochran's Q test is used to detect heterogeneity. The MR-Egger intercept test generates a non-zero intercept, indicating the presence of directional pleiotropy [35]. The MR Pleiotropy Residual Sum and Outlier (MR-PRESSO) test is used to detect potential outliers and provides corrected estimates of MR to account for horizontal pleiotropy after removing such outliers [36]. Leave-one-out analysis checks whether MR estimates are driven by individual SNPs. Additionally, we employed the Steiger test to mitigate the impact of reverse causation [37, 38]. For significant results determined by IVW (*p* < 0.05), we use the PhenoScanner online tool to search for the second phenotypes of genetic variants, including body mass index, obesity, smoking, drinking, neuropsychiatric disease, hypertension, and hyperlipemia, to evaluate whether these MR estimates are overturned by potential confounding factors [39, 40]. These tests have different assumptions but are useful for explaining other causal pathways apart from the hypothesized pathway [34]. Multiple comparisons are corrected by the Bonferroni method. The meaningful and nominally significant thresholds were set at *p* < 0.05/138 = 0.0004 and *p* < 0.05, respectively, out of 138 tests conducted. The analysis was performed using the TwoSampleMR package (version 0.5.6) in R software (version 4.2.1).

### Causal genomic loci and enrichment analysis

To further explore the potential mechanisms underlying cortical structural changes mediated by MS, we examined gene sets with distinct causal effects (increasing or decreasing TH and SA) on cortical structure. These sets were identified through a series of sensitivity tests and filtered MR analysis results. We then performed Gene Ontology (GO) and Kyoto Encyclopedia of Genes and Genomes (KEGG) pathway enrichment analysis on these gene sets. The analysis was completed using the clusterProfiler package (version v4.4.4) and the biomaRt package (version 2.52.0) in R software (version 4.2.1).

## Results

### MR analysis and sensitivity analysis

**Fig. 1:**
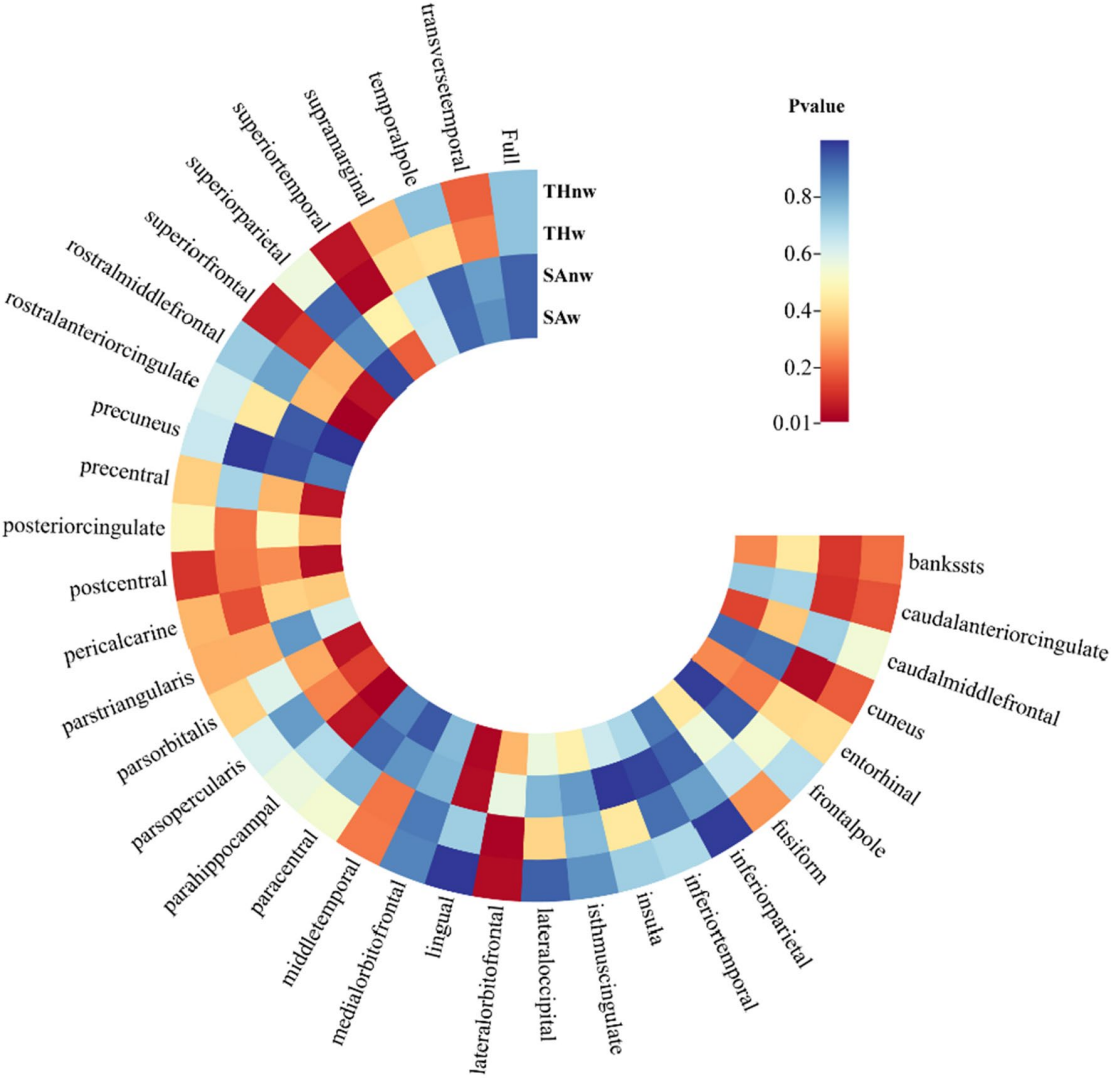
IVW estimates from multiple sclerosis on brain cortical structure. *IVW* the inverse-variance weighted method with random effects, *TH* thickness, *SA* surface area, *w* whole-brain weighted, *nw* whole-brain unweighted

**Fig. 2:**
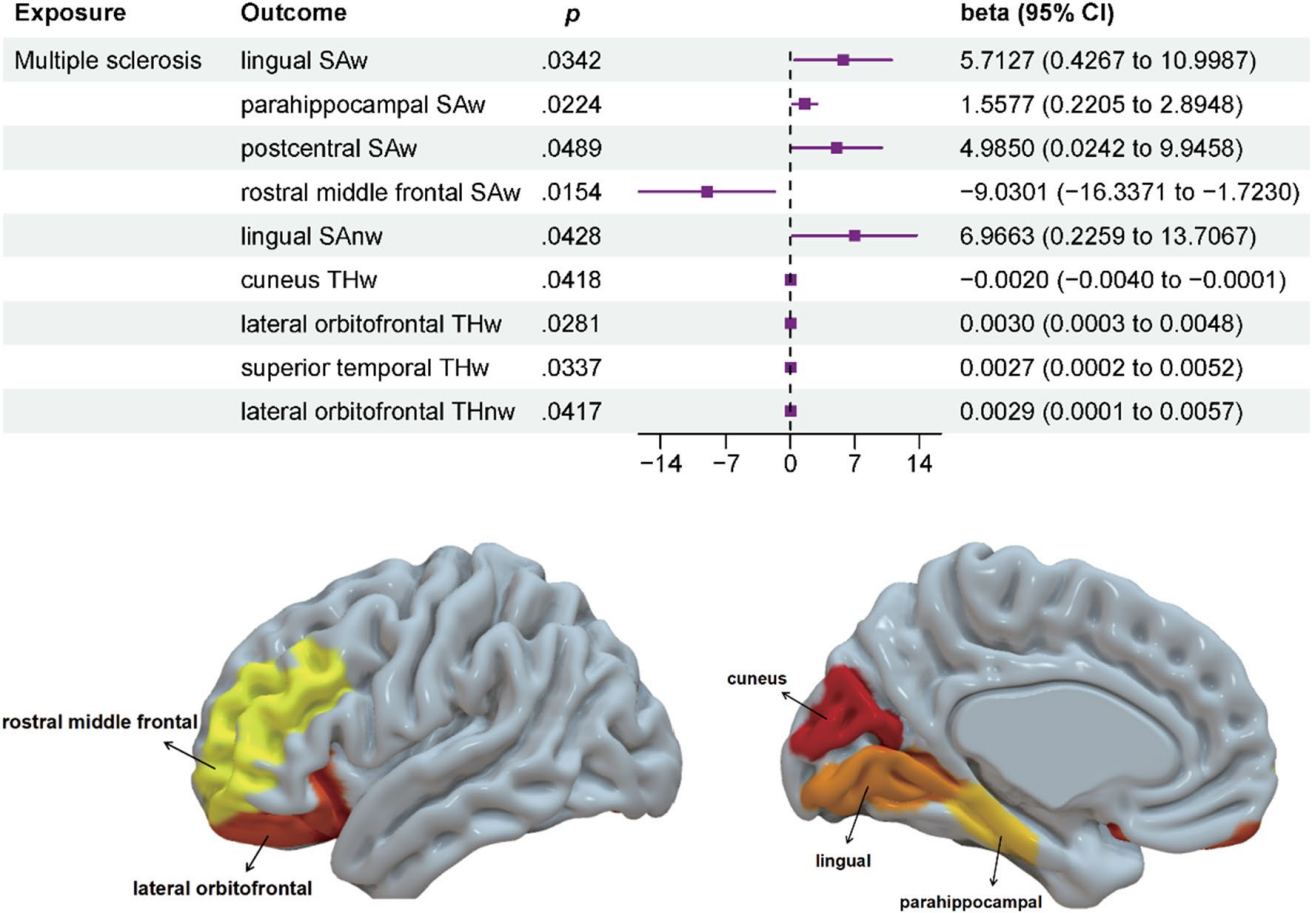
MR estimation of positive primary screening for IVW. *MR* Mendelian randomization, *IVW* the inverse-variance weighted method with random effects, *p* P-value derived from the inverse-variance weighted (IVW) method, *TH* thickness, *SA* surface area, *w* whole-brain weighted, *nw* whole-brain unweighted, *CI* confidence interval

Out of a total of 138 MR analyses, we used the IVW method to preliminarily establish nominal causal associations between MS and cortical structure in 9 brain regions. These regions include lingual SAw, parahippocampal SAw, postcentral SAw, rostral middle frontal SAw, lingual SAnw, cuneus THw, lateral orbitofrontal THw, superior temporal THw, and lateral orbitofrontal THnw (Figs. 1, 2).

Sensitivity analysis showed that the MR-Egger intercept was removed in the lingual SAnw due to the detection of directional pleiotropy (intercept = − 2.51, se = 1.19, *p* = 0.04). Directional pleiotropy was not detected in the remaining MR estimates (MR-Egger intercept *pvalue* > 0.05, Additional file 1: Table S1). Although heterogeneity was detected in the superior temporal THw (Cochran's Q = 68.52, *p* = 0.01), the random effects IVW method accepted the presence of heterogeneity. The MR-PRESSO analysis confirmed the presence of potential outliers in the superior temporal THw region, but their identification was not feasible (*p* = 0.004). However, no outliers were detected in the other MR results (MR-PRESSO *pvalue* > 0.05, Additional file 1: Table S1). The remaining four supplemental methods for MR estimation are shown in Table S2. Leave-one-out analysis showed that none of the MR estimates were driven by a single SNP (Additional file 1: Figs. S1–S9). All MR estimates passed the Steiger test, maintaining consistency with the previous results.

In addition, we further conducted sensitivity analyses using the PhenoScanner tool for the remaining MR estimates. We found that rs7923837 and rs11554159 were associated with body mass index, rs8070345 with frequency of alcohol consumption, rs11154801 with schizophrenia, and rs2857700 with Parkinson's disease, total cholesterol, and self-reported hypertension. After removing the SNPs associated with these risk factors, the MR estimates for postcentral SAw and superior temporal THw were no longer consistent with the previous ones (*pvalue* > 0.05, Additional file 1: Table S3).

Therefore, after a series of sensitivity analyses, only six surviving MR results suggested a causal relationship between MS and cortical structure, including lingual SAw (*p* = 0.0342, beta (se) = 5.7127 (2.6969)), parahippocampal SAw (*p* = 0.0224, beta (se) = 1.5577 (0.6822)), rostral middle frontal SAw (*p* = 0.0154, beta (se) =  − 9.0301 (3.7281)), cuneus THw (*p* = 0.0418, beta (se) =  − 0.0020 (0.0010)), lateral orbitofrontal THw (*p* = 0.0281, beta (se) = 0.0025 (0.0010)), and lateral orbitofrontal THnw (*p* = 0.0417, beta (se) = 0.0029 (0.0014)) (Fig. 2). It is noteworthy that these MR estimates were limited to regional functional levels, and there was no evidence to support a potential causal relationship between MS and global cortical TH or SA (Fig. 1).

### Causal loci and enrichment analysis

**Fig. 3:**
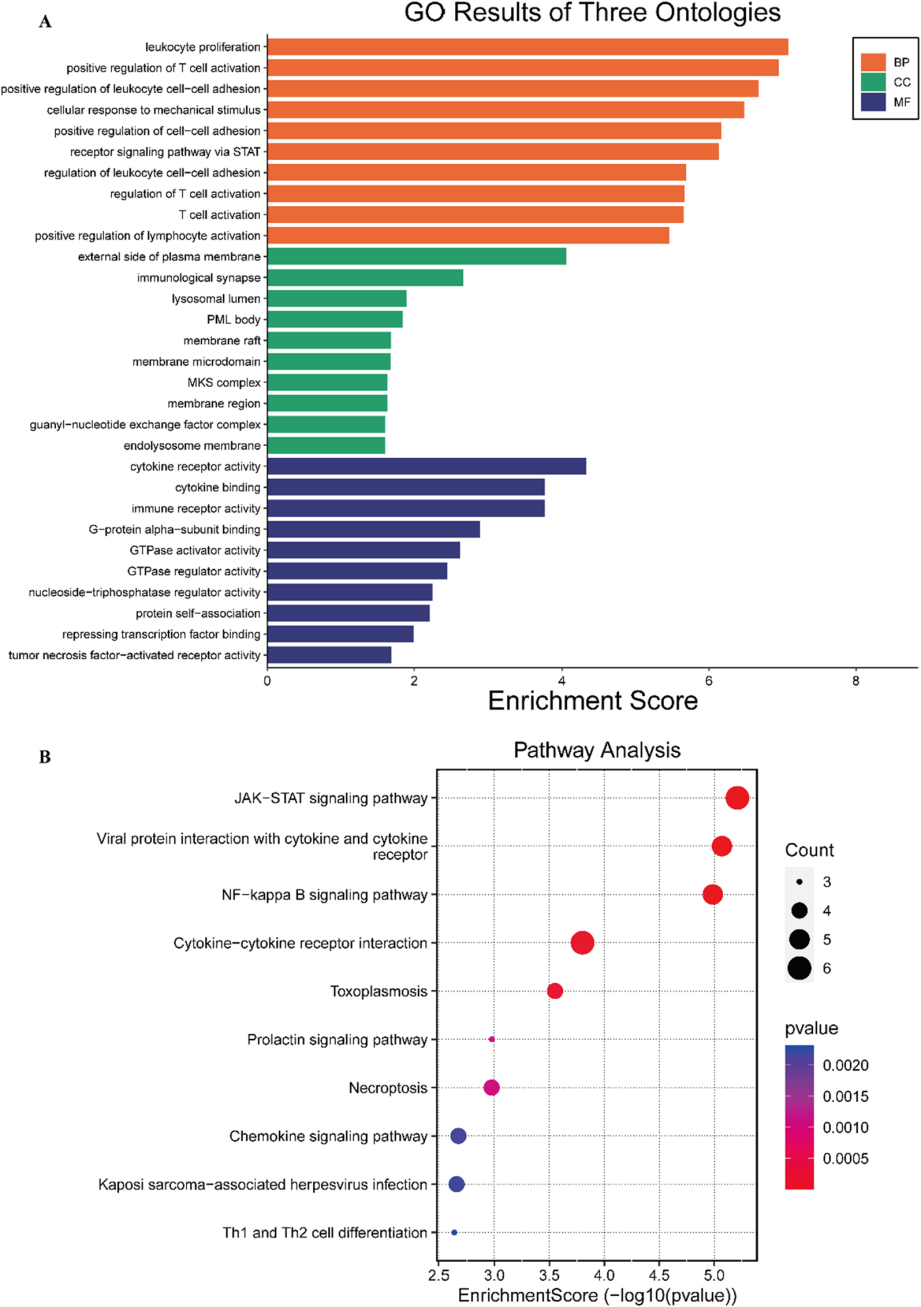
Enrichment analysis of causal SNPs. **A** SNPs: single nucleotide polymorphisms; Gene Ontology (GO) analysis results; **B** Kyoto Encyclopedia of Genes and Genomes (KEGG) analysis results

Although MS has different effects on the TH and SA of the cerebral cortex, we found that the gene sets underlying the causal relationship between MS and brain morphology were completely overlapping. A total of 41 genes were implicated in the causal link between MS and cortical structure (Additional file 1: Table S4). Notably, 13 key genes including ELMO1, SOCS1, STAT3, STAT4, IL7R, IL22RA2, TNFRSF1A, TNFSF14, IL2RA, TNFAIP3, BCL10, MAPK3, CD86, CXCR5, and LTBR were found to regulate the changes in cortical structure caused by MS (Additional file 1: Fig. S10). GO analysis revealed that these pathways were mainly enriched in leukocyte proliferation, positive regulation of T cell activation, positive regulation of leukocyte cell–cell adhesion, and receptor signaling pathways via STAT (Fig. 3A). KEGG analysis showed that these pathways were mainly involved in JAK-STAT signaling, NF-kappa B signaling, cytokine-cytokine receptor interaction, and prolactin signaling pathways (Fig. 3B).

## Discussion

To our knowledge, this is the first MR study investigating the causal relationship between MS and brain structure. We implemented a large-scale GWAS summary data analysis on MS and brain structure, ultimately discovering nominal associations between MS and 6 cortical structures, including decreased rostral middle frontal SAw, cuneus THw, and increased lingual SAw, parahippocampal SAw, lateral orbitofrontal THw, and lateral orbitofrontal THnw. Sensitivity analysis strengthened the robustness of our MR estimates.

Our MR analysis suggested that MS primarily affects cortical structures concentrated in the frontal and temporal lobes, partially overlapping with previous observational studies, although the effects were not entirely consistent. Previous studies have demonstrated atrophy in the precuneus and cuneus regions in MS patients [41, 42], and our findings indicated comparable declines in the TH of the cuneus region. Furthermore, we identified a causal association between MS and increased lingual SA. While most visual impairments in MS are due to optic neuritis [43], these regions are located in the occipital lobe and are closely related to vision, which may explain some of the mechanisms underlying visual impairments in MS patients. Unfortunately, the brain structure GWAS by Grasby et al. did not include the optic nerve [27], so we were unable to determine the causal effect of MS on the optic nerve in the current study.

Furthermore, we presented compelling evidence supporting a causal association between MS and increased SA in the parahippocampal gyrus, elevated TH in the lateral orbitofrontal cortex, and decreased SA in the rostral middle frontal region. Accumulating evidence has suggested a close association between the parahippocampal gyrus and olfactory and cognitive impairments [44–46], with reports of 20 to 45% of MS patients experiencing olfactory dysfunction [47]. Moreover, the parahippocampal gyrus is directly connected to multiple regions of the prefrontal cortex [44], and olfactory dysfunction may be associated with damage to the subfrontal and temporal lobes [48]. Steenwijk et al. observed that the pattern of cortical thickness changes in individuals with MS primarily involves bilateral temporal poles and insular cortex [9]. Several observational studies have reported cortical thinning in the frontal and temporal lobes of MS patients [10, 49–51]. Our MR estimates similarly suggest damage to the temporal and frontal lobes, especially a decrease in rostral middle frontal SA. Therefore, it is reasonable to speculate that damage to the olfactory pathway from the temporal lobe to the frontal cortex, especially the orbitofrontal cortex, may contribute to the olfactory decline in MS patients [46, 47]. It is worth noting that the parahippocampal cortex is associated with various cognitive processes, including visual spatial processing and episodic memory [44, 45]. Although our results suggest an association between MS and the parahippocampal cortex, further research is needed to determine whether MS patients experience neurobehavioral symptoms through the impact on the parahippocampal gyrus.

Our findings of increased cortical SA or TH in some regions, though unexpected, are supported by limited observational studies. A magnetic resonance imaging study of patients with clinically isolated syndrome suggestive of MS within 2 years of onset showed increased volume in frontal, parietal, temporal, and cerebellar gray matter regions at 3 months [52]. Another study focusing on mildly disabled patients with RRMS showed increased TH in two visual cortical areas, the left hemisphere's inferior occipital gyrus and the right hemisphere's cuneus [53]. The researchers speculated that cortical reorganization in adjacent brain regions and compensatory mechanisms recruited by increased cortical networks could reduce disease activity and even result in compensatory hypertrophy or brain edema, leading to increased cortical volume [40, 53]. Furthermore, unconventional cortical changes have been observed in other neurological diseases, such as larger cortical SA in early-stage Parkinson's disease patients compared to controls [54]. An ENIGMA study showed a correlation between smoking and increased brain structural changes [55]. Thus, MS may not be limited to the pattern of gray matter atrophy alone. However, the underlying mechanisms are not fully understood and require further clarification. Additionally, we noted inconsistent trends in cortical SA and TH morphology, which may be attributed to their different anatomical developmental patterns [40].

Research has confirmed that alterations in the function of regulatory T cells and/or effector B cells and T cells disrupt peripheral tolerance mechanisms in patients with MS, thereby promoting the generation of pro-inflammatory mediators such as cytokines. The communication between the peripheral immune system and the central nervous system, facilitated by messenger molecules like cytokines, leads to neuroinflammation and immune responses in the brain and spinal cord regions [56]. Through causal gene enrichment analysis, we identified pathways mediating cortical changes in MS that predominantly cluster in leukocyte cell-related pathways, cytokine-cytokine receptor interaction, JAK-STAT signaling pathway, and NF-kappa B signaling pathway. These pathways are intricately linked to various immune and inflammatory processes [56–59]. Simultaneously, gray matter damage is closely associated with peripheral-induced neuroinflammation and immune responses [60–62]. Given these findings, we speculate that the structural abnormalities in the brains of MS patients are primarily driven by immune and inflammatory pathways. Our findings suggest that the current disease-modifying treatment strategy [56], primarily focused on anti-inflammatory approaches, is well-founded. This is further supported by certain drugs, such as selective sphingosine 1-phosphate receptor subtype 1 [61]. Additionally, considering the successful application of Janus kinase inhibitors (Jakinibs) in the treatment of rheumatic diseases [59], we propose that Jakinibs could serve as candidate drugs for treating cortical changes in MS, pending further research to confirm this hypothesis.

The present study should be considered in light of its strengths and limitations. Our study has several strengths. Firstly, we employed a two-sample MR design, which minimizes confounding factors and reverses causation that is inevitable in observational studies. Secondly, we strengthened the statistical power by using a stringent P-value threshold and F-statistics. Thirdly, sensitivity analyses provided no evidence of horizontal pleiotropy, reinforcing the robustness of our MR estimates. However, our study has several limitations. Firstly, our study population was limited to individuals of European ancestry, which restricts the generalizability of our results to other populations. Future studies in other populations are warranted. Secondly, a large body of research has focused on RRMS and PMS patients, whereas our MR estimates were based on summary-level data and do not provide more specific subtype information. This implies that stratified analyses may lead to inconsistent conclusions. However, researchers have increasingly recognized that different phenotypes of MS may represent different courses of the same disease [63], making our MR analysis appropriate from that perspective. Thirdly, although the causality between MS and cortical structure is suggestive, Bonferroni correction is considered conservative [64].

In summary, we provide robust evidence supporting a causal relationship between genetic proxies of MS and cortical structure. Enrichment analysis suggests that the pathways mediating brain morphology abnormalities in MS patients are mainly related to immune and inflammation-driven pathways. Further research is needed to validate these findings.

### Supplementary Information

**Additional file 1: Table S1. **Results of sensitivity analysis. **Table S2. **Results of MR-Egger,weighted median,simple mode,weighted mode methods. **Table S3. **IVW results after removing confounders. **Table S4. **Nearest genes from causal SNPs. **Figure S1. **Leave-one-out analysis of lingual SAw. **Figure S2.** Leave-one-out analysis of parahippocampal SAw. **Figure S3.** Leave-one-out analysis of postcentral SAw. **Figure S4.** Leave-one-out analysis of rostral middle frontal SAw. **Figure S5.** Leave-one-out analysis of lingual SAnw. **Figure S6.** Leave-one-out analysis of cuneus THw. **Figure S7.** Leave-one-out analysis of lateral orbitofrontal THw. **Figure S8.** Leave-one-out analysis of superior temporal THw. **Figure S9.** Leave-one-out analysis of lateral orbitofrontal THnw. **Figure S10.** Network diagram for pathway analysis.

## Data Availability

Data supporting the findings of this study are available from the article/additional material.

## Abbreviations

MS: Multiple sclerosis
RRMS: Relapsing-remitting multiple sclerosis
PMS: Progressive multiple sclerosis
TH: Thickness
SA: Surface area
MR: Mendelian randomization
SNPs: Single nucleotide polymorphisms
IVs: Instrumental variables
GWAS: Genome-wide association study
IMSGC: International Multiple Sclerosis Genetics Consortium
ENIGMA: Enhancing NeuroImaging Genetics through Meta-Analysis Consortium
IVW: The inverse-variance weighted method with random effects
MR-PRESSO: MR Pleiotropy Residual Sum and Outlier test
GO: Gene Ontology
KEGG: Kyoto Encyclopedia of Genes and Genomes
