# Supplementary material for: Causal relationship between multiple sclerosis and cortical structure: a Mendelian randomization study
Source: J Transl Med. 2024 Jan 20;22:83. doi: 10.1186/s12967-024-04892-7 (PMC10800041; doi:10.1186/s12967-024-04892-7)

**Additional Information**

**Causal relationship between multiple sclerosis and cortical structures: a Mendelian randomization study**

Dongren Sun^1^, Rui Wang^1^, Qin Du^1^, Ying Zhang^1^, Hongxi Chen^1^, Ziyan Shi^1^, Xiaofei Wang^1,*^, Hongyu Zhou^1,*^

^1^Department of Neurology, West China Hospital, Sichuan University, Guo Xuexiang #37, Chengdu 610041, China.

^*^Xiaofei Wang and Hongyu Zhou contributed equally to this work.

**Content:**

**Table S1** Results of sensitivity analysis.

**Table S2** Results of MR-Egger,weighted median,simple mode,weighted mode methods.

**Table S3** IVW results after removing confounders.

**Table S4** Nearest genes from causal SNPs.

**Figure S1**  Leave-one-out analysis of lingual SAw.

**Figure S2**  Leave-one-out analysis of parahippocampal SAw.

**Figure S3** Leave-one-out analysis of postcentral SAw.

**Figure S4** Leave-one-out analysis of rostral middle frontal SAw.

**Figure S5** Leave-one-out analysis of lingual SAnw.

**Figure S6**  Leave-one-out analysis of cuneus THw.

**Figure S7** Leave-one-out analysis of lateral orbitofrontal THw.

**Figure S8** Leave-one-out analysis of superior temporal THw.

**Figure S9** Leave-one-out analysis of lateral orbitofrontal THnw.

**Figure S10** Network diagram for pathway analysis.

Notes: IVW: the inverse-variance weighted method with random effects; SNPs: single nucleotide polymorphisms; THw: cortical thickness with global weighted; THnw: cortical thickness without global weighted; SAw: cortical surface area with global weighted; SAnw: cortical surface area without global weighted.

**Table S1** Results of sensitivity analysis.

Notes: THw: cortical thickness with global weighted; THnw: cortical thickness without global weighted; SAw: cortical surface area with global weighted; SAnw: cortical surface area without global weighted. MR-PRESSO: Mendelian randomization pleiotropy residual sum and outlier test.

**Table S2** Results of MR-Egger,weighted median,simple mode,weighted mode methods.

Notes: *pvalue*: P-value derived from the inverse-variance weighted (IVW) method with random effects. THw: cortical thickness with global weighted; THnw: cortical thickness without global weighted; SAw: cortical surface area with global weighted; SAnw: cortical surface area without global weighted.

**Table S3** IVW results after removing confounders.

| **Exposure** | **Outcome** | ***pvalue*** | **beta** | **95% Confidence interval** | |
| --- | --- | --- | --- | --- | --- |
| MS | lingual SAw | 0.041 | 6.5932 | 0.2680 | 12.9184 |
|  | parahippocampal SAw | 0.041 | 6.5932 | 0.2680 | 12.9184 |
|  | postcentral SAw | 0.056 | 5.7818 | -0.1492 | 11.7128 |
|  | rostral middle frontal SAw | 0.001 | -14.3294 | -22.9088 | -5.7501 |
|  | lingual SAnw | 0.719 | 1.4455 | -6.4416 | 9.3327 |
|  | cuneus THw | 0.041 | -0.0024 | -0.0047 | -0.0001 |
|  | lateral orbitofrontal THw | 0.006 | 0.0035 | 0.0010 | 0.0060 |
|  | superior temporal THw | 0.463 | 0.0010 | -0.0017 | 0.0037 |
|  | lateral orbitofrontal THnw | 0.005 | 0.0045 | 0.0014 | 0.0076 |

Notes: IVW: the inverse-variance weighted method with random effects; MS: multiple sclerosis. *pvalue*: P-value derived from the inverse-variance weighted (IVW) method with random effects. THw: cortical thickness with global weighted; THnw: cortical thickness without global weighted; SAw: cortical surface area with global weighted; SAnw: cortical surface area without global weighted.

**Table S4** Nearest genes from causal SNPs.

Notes: THw: cortical thickness with global weighted; THnw: cortical thickness without global weighted; SAw: cortical surface area with global weighted; SAnw: cortical surface area without global weighted.

**Figure S1**  Leave-one-out analysis of lingual SAw.


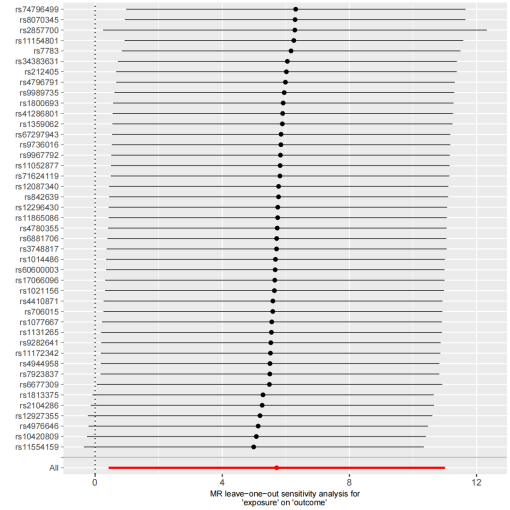


Notes: SAw: cortical surface area with global weighted.

**Figure S2**  Leave-one-out analysis of parahippocampal SAw.


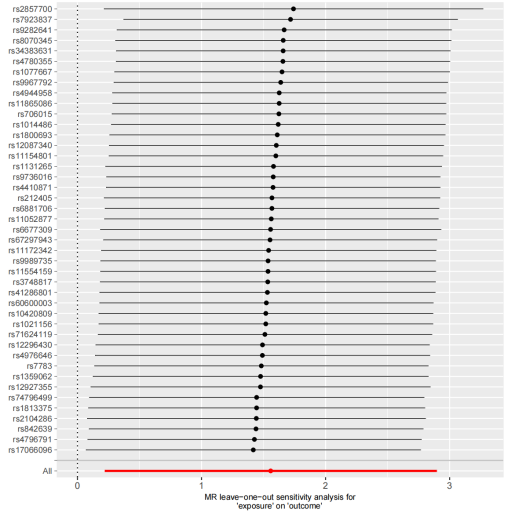


Notes: SAw: cortical surface area with global weighted.

**Figure S3** Leave-one-out analysis of postcentral SAw.


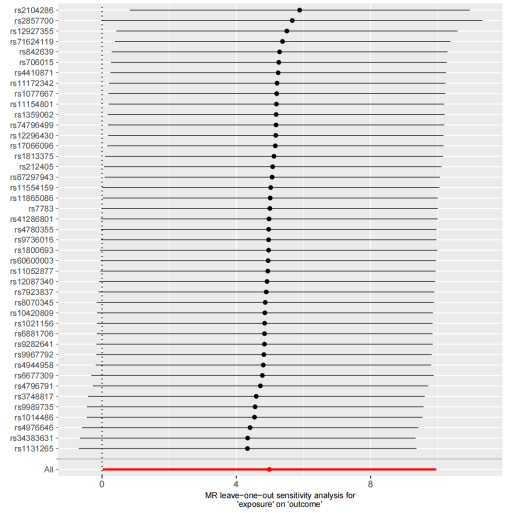


Notes: SAw: cortical surface area with global weighted.

**Figure S4** Leave-one-out analysis of rostral middle frontal SAw.


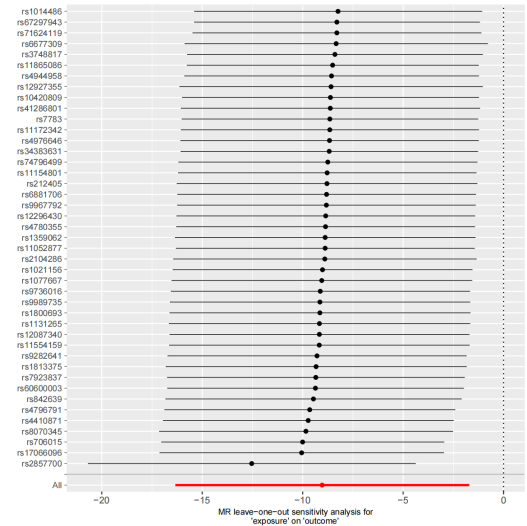


Notes: SAw: cortical surface area with global weighted.

**Figure S5** Leave-one-out analysis of lingual SAnw.


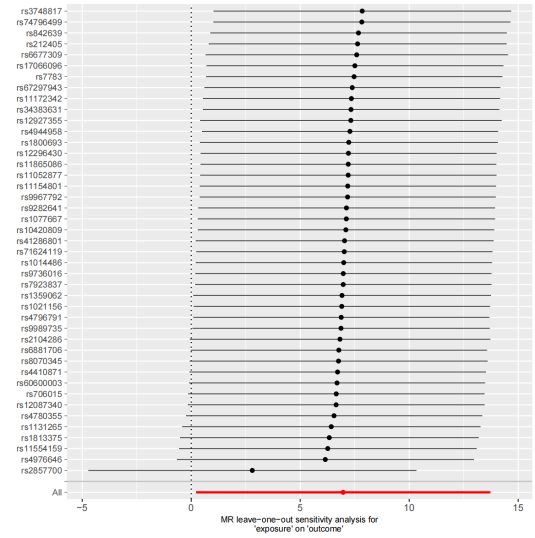


Notes: SAnw: cortical surface area without global weighted.

**Figure S6**  Leave-one-out analysis of cuneus THw.


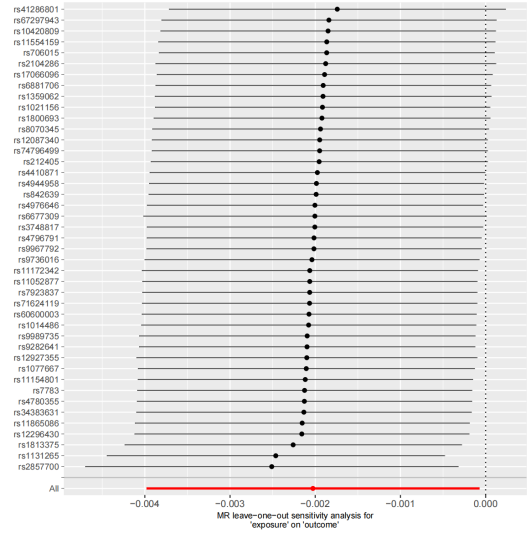


Notes: THw: cortical thickness with global weighted.

**Figure S7** Leave-one-out analysis of lateral orbitofrontal THw.


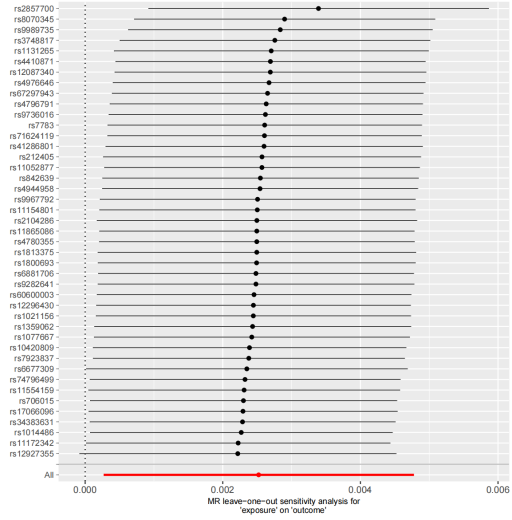


Notes: THw: cortical thickness with global weighted.

**Figure S8** Leave-one-out analysis of superior temporal THw.


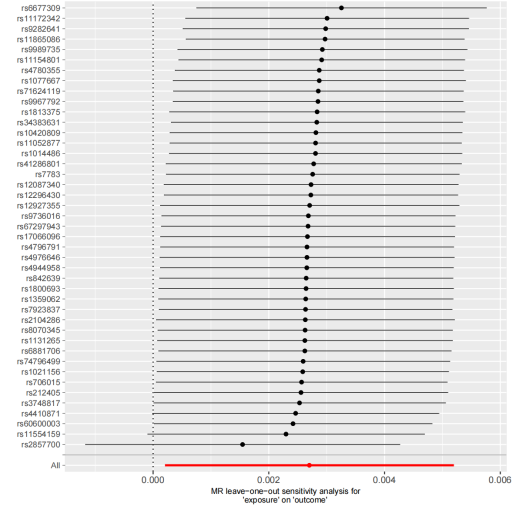


Notes: THw: cortical thickness with global weighted.

**Figure S9** Leave-one-out analysis of lateral orbitofrontal THnw.


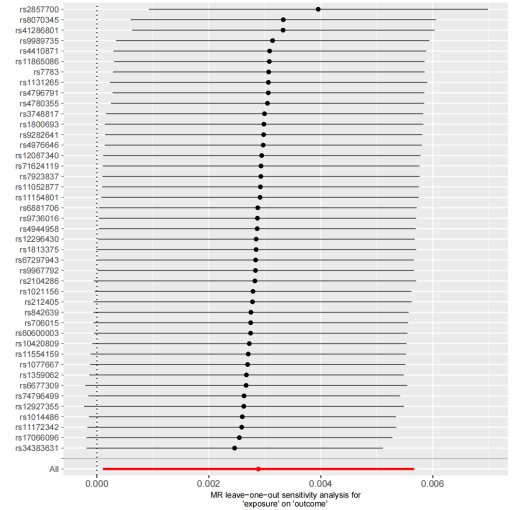


Notes: THnw: cortical thickness without global weighted.

**Figure S10**  Network diagram for pathway analysis.


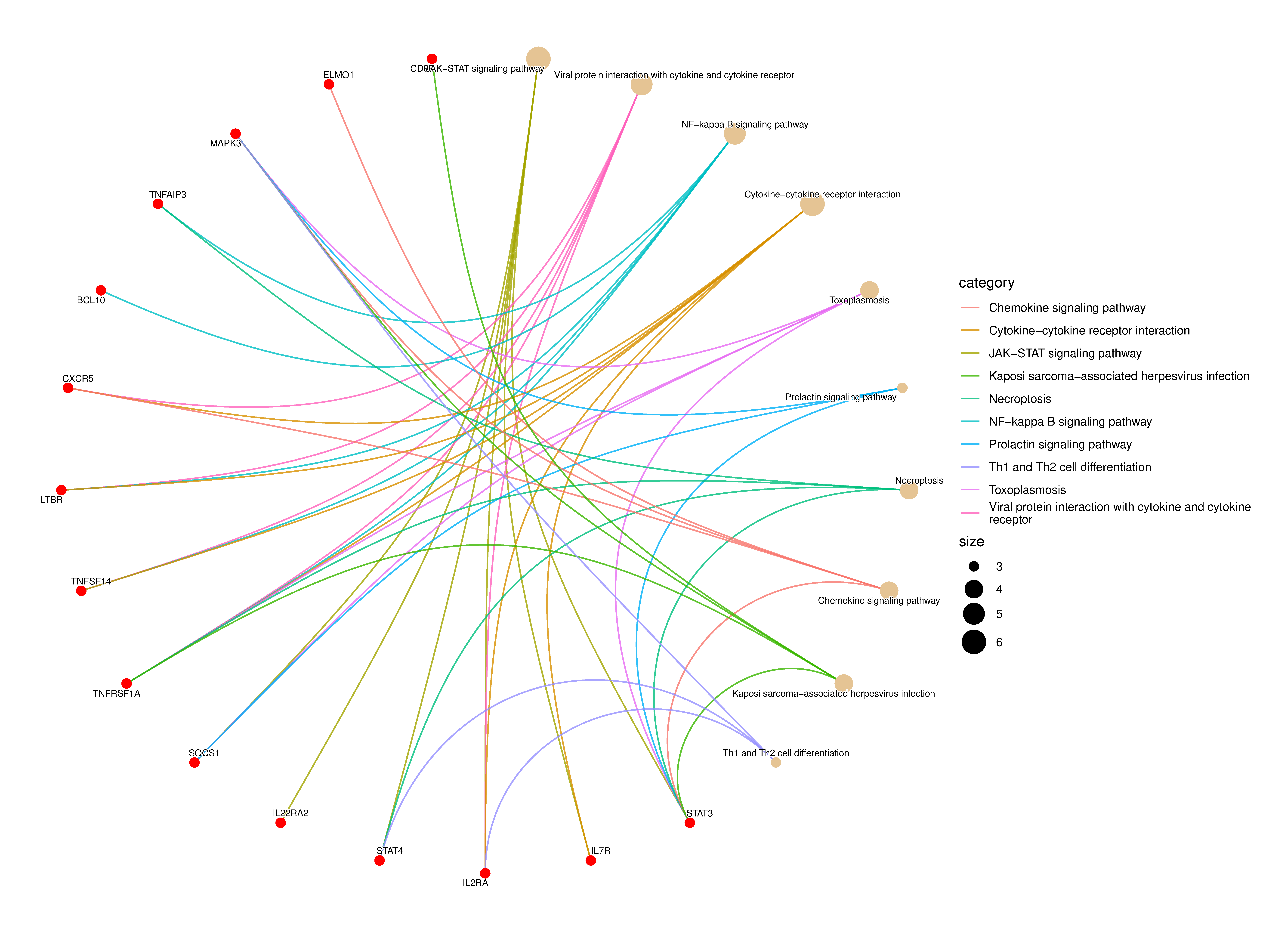

Supplement: Supplementary file 1 — Additional file 1: Table S1. Results of sensitivity analysis. Table S2. Results of MR-Egger,weighted median,simple mode,weighted mode methods. Table S3. IVW results after removing confounders. Table S4. Nearest genes from causal SNPs. Figure S1. Leave-one-out analysis of lingual SAw. Figure S2. Leave-one-out analysis of parahippocampal SAw. Figure S3. Leave-one-out analysis of postcentral SAw. Figure S4. Leave-one-out analysis of rostral middle frontal SAw. Figure S5. Leave-one-out analysis of lingual SAnw. Figure S6. Leave-one-out analysis of cuneus THw. Figure S7. Leave-one-out analysis of lateral orbitofrontal THw. Figure S8. Leave-one-out analysis of superior temporal THw. Figure S9. Leave-one-out analysis of lateral orbitofrontal THnw. Figure S10. Network diagram for pathway analysis. [file 12967_2024_4892_MOESM1_ESM.docx]
